# Traffic lights for retinoids in oncology: molecular markers of retinoid resistance and sensitivity and their use in the management of cancer differentiation therapy

**DOI:** 10.1186/s12885-018-4966-5

**Published:** 2018-11-01

**Authors:** Viera Dobrotkova, Petr Chlapek, Pavel Mazanek, Jaroslav Sterba, Renata Veselska

**Affiliations:** 1 Laboratory of Tumor Biology, Department of Experimental Biology, Faculty of Science, Masaryk University, Kotlarska 2, 61137 Brno, Czech Republic; 2 International Clinical Research Center, St. Anne’s University Hospital, Pekarska 53, 65691 Brno, Czech Republic; 3 Department of Pediatric Oncology, University Hospital Brno and Faculty of Medicine, Masaryk University, Cernopolni 9, 61300 Brno, Czech Republic

**Keywords:** Retinoids, Cell differentiation, Retinoid resistance, Retinoid sensitivity, Predictive biomarkers, Acute myeloid leukemia, Pancreatic ductal adenocarcinoma, Breast carcinoma, Neuroblastoma

## Abstract

For decades, retinoids and their synthetic derivatives have been well established anticancer treatments due to their ability to regulate cell growth and induce cell differentiation and apoptosis. Many studies have reported the promising role of retinoids in attaining better outcomes for adult or pediatric patients suffering from several types of cancer, especially acute myeloid leukemia and neuroblastoma. However, even this promising differentiation therapy has some limitations: retinoid toxicity and intrinsic or acquired resistance have been observed in many patients. Therefore, the identification of molecular markers that predict the therapeutic response to retinoid treatment is undoubtedly important for retinoid use in clinical practice. The purpose of this review is to summarize the current knowledge on candidate markers, including both genetic alterations and protein markers, for retinoid resistance and sensitivity in human malignancies.

## Introduction

Defective or aberrant cell differentiation is a hallmark of many human malignancies. The initial step in an aberrant tumor cell phenotype involves various mutations that alter signaling pathways, epigenetic modifiers, and transcription factors, leading to the deregulated expression of proteins required for cell differentiation.

During the 1970s and 1980s, as an elegant alternative to killing cancer cells by cytotoxic therapies, several scientific achievements popularized the strategy of inducing malignant cells to overcome differentiation inhibition and to enter apoptotic pathways [1]. The initial preclinical results proved to be very promising and fueled hope for the development of a new approach in cancer treatment called “differentiation therapy” [2].

In general, differentiation therapy aims to reactivate the endogenous differentiation program in transformed cells to resume the mutation process and eliminate the tumor phenotype. Thus, this strategy offers the prospect of a less aggressive treatment that limits damage to the normal cells in the organism.

## Natural and synthetic retinoids in anticancer treatment

Retinoids, i.e., natural and synthetic vitamin A derivatives, have been studied for decades in clinical trials due to their established role in regulating cell growth, differentiation and apoptosis. Retinoids are key compounds in biological differentiation therapy. Retinoids have critical functions in many aspects of human biology: at the cellular level, they control cell differentiation, growth, and apoptosis [3]. Several biologically active vitamin A derivatives, namely, all-*trans* retinoic acid (ATRA), 9-*cis* retinoic acid (9-*cis-*RA), and 13-*cis* retinoic acid (13-*cis*-RA), have been tested for potential use in cancer therapy and chemoprevention [4–7]. The most effective clinical use of ATRA was demonstrated in acute promyelocytic leukemia (APL) treatment [8]. Additional studies have indicated that 13-*cis*-RA is beneficial in high-risk neuroblastoma (NBL) treatment after bone marrow transplantation, suggesting that retinoids may play an adjuvant therapeutic role in the management of minimal residual disease [9]. List of all human malignancies, for which the clinical treatment with retinoids was already tested, is given in the Table 1.

**Table 1:** Overview of the human cancer types treated with retinoids in clinical studies

Type of cancer	Retinoid	Type of treatment	Reference
Acute myeloid leukemia	ATRA	Trial Phase III	[101]
Acute promyelocytic leukemia	ATRA	Trial Phase IV	[8]
B-cell lymphoma	Fenretinide	Trial Phase II	[102]
Breast carcinoma	ATRA	Observational study	[103]
Cervical carcinoma	13-*cis*-RA	Trial Phase II	[104]
Cutaneous T-cell lymphoma	Bexarotene	Trial Phase II-III	[105]
Ewing’s sarcoma	Fenretinide	Trial Phase I	[106]
	13-*cis*-RA	Observational study	[107]
Glioblastoma multiforme	13-*cis*-RA	Trial Phase II	[108]
Gliomas	13-*cis*-RA	Trial Phase III	[109]
	Fenretinide	Trial Phase II	[110]
Hepatocellular carcinoma	Polyprenoic acid	Observational study	[111]
Mantle cell lymphoma	Fenretinide	Trial Phase II	[102]
Medulloblastoma	Fenretinide	Trial Phase I	[106]
	13-*cis*-RA	Observational study	[107]
Multiple myeloma	ATRA	Trial Phase II	[112]
Neuroblastoma	13-*cis*-RA	Observational study	[107]
		Trial Phase I	[9]
	Fenretinide	Trial Phase I	[106]
Non-small lung cancer	ATRA	Trial Phase II	[113]
Osteosarcoma	13-*cis*-RA	Observational study	[107]
	Fenretinide	Trial Phase I	[106]
Ovarian carcinoma	Fenretinide	Trial Phase II	[114]
Pancreatic carcinoma	ATRA	Trial Phase I	[115]
Papillary thyroid cancer	13-*cis*-RA	Observational study	[116]
Prostate carcinoma	Fenretinide	Trial Phase II	[117]
Renal carcinoma	Fenretinide	Trial Phase II	[118]
Small cell lung cancer	Fenretinide	Trial Phase II	[119]
Squamous cell carcinoma	13-*cis*-RA	Case series trial	[120]
T-cell malignancies	13-*cis*-RA	Phase II	[121]
Wilm’s tumor	Fenretinide	Phase I	[106]

Nevertheless, vitamin A-associated toxicity involving liver and lipid alterations, dry skin, teratogenicity, bone and connective tissue damage substantially limits the long-term administration of natural retinoids. Both ATRA and 13-*cis* RA are pan-RAR activators, which can explain their large negative side effects. For these reasons, the modification of several functional groups has produced new, synthetic retinoids that have increased chemoprevention efficacy and reduced toxicity compared with these parameters in other natural retinoids. These modifications include the substitution of benzoic acid with aromatic rings or can change their solubility in water, for example. Fenretinide (N-(4-hydroxyphenyl) retinamide, 4-HPR) has been discussed as an effective cancer treatment, especially due to its pro-apoptotic and anti-angiogenic effects even in ATRA-resistant cell lines and with minor side-effects profile [10]. Bexarotene is a synthetic retinoid that is approved by the European Medicines Agency to treat skin manifestations of advanced-stage cutaneous T-cell lymphoma in adult patients refractory to at least one systemic treatment [11]. Several studies have suggested that bexarotene is an effective anticancer treatment that is able to decrease proliferation and promote apoptosis in cells expressing retinoid X receptors (RXRs) [12, 13]. A very recent study described synthesis of a novel retinoid WYC-209, which abrogates growth of melanoma tumor-repopulating cells and inhibits lung metastases in vivo, showing minimal toxicity on non-tumor cells [14].

When it comes to synthetic RA analogues that are still being synthesized and tested, the biggest disadvantage of such new compounds is undoubtedly the lack of information about their long-term effects on human body.

## Mechanisms of retinoid resistance

Biological retinoid activity is based on the binding of retinoids to specific nuclear receptors (retinoic acid receptors (RARs) bind retinoic acid and RXRs bind retinoids) that act as inducible transcription factors. When activated, these nuclear receptors form RXR-RAR heterodimers or RXR-RXR/RAR-RAR homodimers that subsequently modulate retinoid-responsive gene expression two ways: (i) by binding to retinoic acid response elements (RAREs) in the promoter regions of target genes or (ii) by antagonizing the enhancer action of other transcription factors, such as AP1 or NF-IL6 [15].

Although pharmacological retinoid doses have been approved by the Food and Drug Administration (FDA) and other regulatory bodies for the treatment of some hematologic malignancies and high-risk NBL, the chemopreventive and therapeutic effects of retinoids in other solid tumors are still unclear. Even in tumors that are treated with retinoids the therapeutic response to the retinoids is often limited to a small proportion of the treated patients [16]. This limited effect is thought to result from retinoid resistance, which is defined as the lack of a tumor cell response to the same pharmacological dose of retinoids that sensitive cells respond to, as evidenced by proliferation arrest or differentiation. Moreover, after retinoid treatment, some carcinomas not only fail to exhibit growth inhibition but instead respond with enhanced proliferation. A clue to this paradoxical behavior was suggested by the finding that retinoic acid and its natural receptor also activate peroxisome proliferator-activated receptor (PPAR) β and δ (PPARβ/δ), which are involved in mitogenic and anti-apoptotic activities [17].

**Fig. 1:**
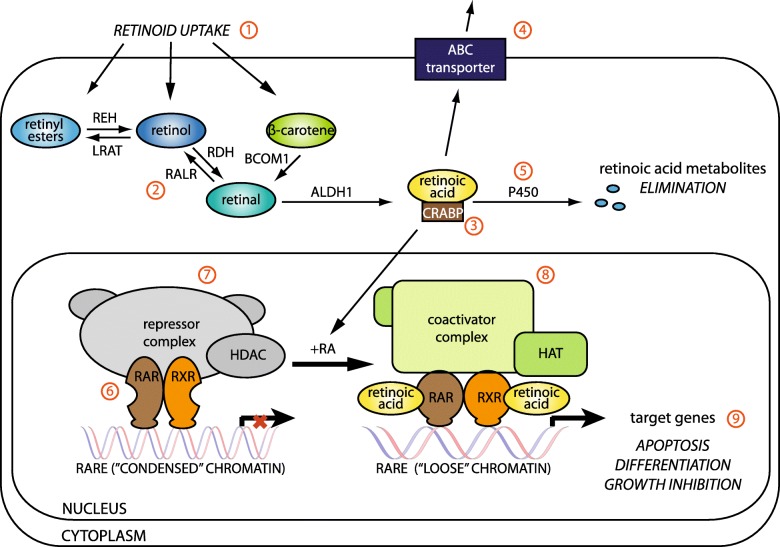
Possible mechanisms of retinoid resistance. Cancer cell retinoid resistance may be caused by several independent mechanisms including (1) decreased retinoid uptake; (2) intracellular retinoid metabolism; (3) altered intracellular retinoid availability due to CRAB protein binding; (4) increased retinoid efflux by ABC transporters; (5) increased retinoid catabolism catalyzed by cytochrome P450; (6) decreased RAR and/or RXR expression; (7) inhibited retinoid-induced transcription by the repressor complex, (8) altered coactivator structure, expression, or activity; (9) altered downstream target gene expression

Many potential mechanisms have been proposed for retinoid resistance (Fig. 1). In general, the cancer cell response to the pharmacological retinoid doses is affected by several mechanisms, including decreased retinoid uptake [18], increased retinoid catabolism by cytochrome P450 [19], active drug efflux by membrane transporters, the downregulated expression of various RAR genes (promoter methylation), the altered expression of coactivators or downstream target genes, and changes in the activities of other signaling pathways [20].

Although retinoid resistance remains problematic in the area of biological anticancer therapy, the discovery of biomarkers that indicate retinoid resistance or sensitivity in each individual patient seems to be important for the recent personalized therapy strategy, which is aimed at identifying of the most effective therapy for individual patients. In the next chapters, we focus on describing the most promising putative biomarkers that predict retinoid resistance or sensitivity in the most relevant cancer types.

## Predictive biomarkers of retinoid resistance

During the past decades, several biomarkers have been identified that can predict the therapeutic response to retinoid treatment in a few human malignancies, including adult leukemia, pancreatic and breast carcinoma and pediatric NBL. These predictive biomarkers are both genetic alterations (typically chromosomal translocations leading to fusion protein expression) and proteins (upregulated or downregulated). In the following parts of this review, we present the recent knowledge concerning these biomarkers in relation to retinoid resistance and sensitivity. An overview of all these biomarkers is given in the Table 2.

**Table 2:** Overview of the candidate biomarkers for predicting the retinoid treatment response in various human malignancies

Putative predictive biomarker	Tumor type	Experimental model	Reference
Biomarkers indicating retinoid resistance			
MN1 overexpression	AML	83 newly diagnosed patients (60 years or older) treated in the trial NCT00151255	[28]
PML-RARA expression	APL	NB4 cell line	[32]
PLZF-RARA+RARA-PLZF expression	APL	Case reports of 6 patients with PLZF-RARA fusion genes with no clinically significant response to ATRA	[42]
IRF2BP2-RARA expression	APL	Case report of 1 patient resistant to ATRA	[44]
STAT5b-RARA expression	APL	Case report of 1 patient resistant to ATRA	[43]
PML L-type splicing variant in E5(−)E6(−) isoform	APL	Short report of 79 de novo patients	[57]
PML V-type splicing variant with spacer between PML-RARA	APL	Sequence analysis of RARα genomic region of 3 patients	[61]
FABP5 overexpression	PDAC	14 patient-derived cell lines	[71]
	BC	MCF-7 cell line	[17]
Truncated RARβ’ isoform expression	BC	MCF-7 cell line	[78]
ERBB2 expression	BC	MCF-7 and HER2/NEU transfected MCF-7 cell lines	[79]
CRABP1 expression	BC	FFPE breast tumor tissue samples, established cell lines	[81]
CRABP2 knockdown	PDAC	14 patient-derived cell lines	[71]
*NF1* knockdown	NBL	Panel of 25 cell lines	[91]
HMGA2 expression	NBL	4 established cell lines	[96]
UNC45 expression	NBL	F9 mouse embryo teratocarcinoma cell line	[100]
Biomarkers indicating retinoid sensitivity			
NuMA-RARA expression	APL	Frozen bone marrow samples	[40]
NPM1-RARA expression	APL	Cultured bone marrow cells from patient harvested at time of relapse	[41]
PLZF-RARA expression	APL	Case report of 62-year-old patient	[54]
RARα receptor overexpression	BC	2 established cell lines, tissue cultures of primary breast tumors, 42 established cell lines	[76, 77]
ZNF423 expression	NBL	Panel of 25 cell lines	[91]
PBX1 expression	NBL	16 established cell lines, 3 independent clinical datasets (ganglioneuromas *n* = 7, low-risk NBL *n* = 11, intermediate-risk NBL *n* = 5)	[88]
HOXC9 expression	NBL	3 established cell lines	[89]

*AML* acute myeloid leukemia, *APL* acute promyelocytic leukemia, *PDAC* pancreatic ductal adenocarcinoma, *BC* breast carcinoma, *NBL* neuroblastoma

### Predictive biomarkers in acute myeloid leukemia

Acute myeloid leukemia (AML) is a heterogenous malignant clonal disease characterized by the accumulation of undifferentiated myeloid blasts, which predisposes patients, especially those with APL-type AML, to overcome impaired differentiation via differentiation-inducing agents, such as granulocyte-colony stimulating factor (GCSF) or ATRA, in addition to conventional chemotherapy [21, 22]. Despite providing high cure rates, such approach is associated with hematologic toxicity as well as with the risk of secondary myeloid neoplasms in approximately 2% of patients. The introduction of arsenic trioxide (ATO) and especially the studies on combined treatment with ATRA plus ATO showed the possibility how to improve the effectiveness of ATRA in APL patients: two large independent randomized trials reported significant improvement in clinical outcome of patients treated with ATRA-ATO if compared with those receiving ATRA only [23, 24].

Studies from the last decade identified *meningioma 1* (*MN1*) as a hematopoietic oncogene with a key role in myeloid leukemogenesis. Based on the gene expression analyses in several hundreds of AML patients, *MN1* overexpression is associated with a poor prognosis in these patients [25–27]. Specifically, 67.4% AML patients had high levels of *MN1* expression if compared with control group and 75% of AML patients with high *MN1* expression were classified as of intermediate risk according cytogenetic risk categories [27]. The MN1 protein seems to have at least two functions: promote self-renewal and proliferation and block cell differentiation [28]. Interestingly, MN1 locates to RAREs and has been implicated as a transcription cofactor in RAR-RXR-mediated transcription [29]. A study on the MN1 expression pattern in AML patients revealed that MN1 overexpression is strongly associated with resistance to ATRA-induced differentiation and cell cycle arrest. In MN1-overexpressing hematopoietic cells, several genes regulated by RARα (*p21, p27*) were repressed and were not upregulated by ATRA treatment [28].

**Fig. 2:**
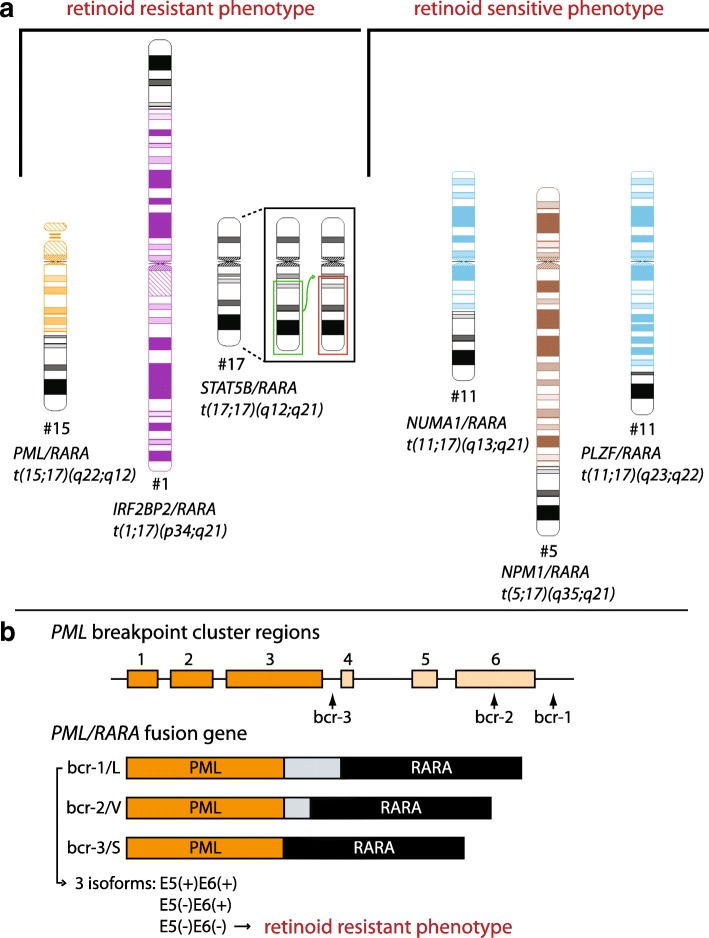
Genetic alterations used as predictive biomarkers for APL patients. **a** Chromosomal translocations between *RARA* and several fusion partners playing an important role in maintaining resistance/sensitivity of APL patients to retinoids [122]. **b** Breakpoint cluster regions (bcr) in *PML* gene resulting in alternative splicing and different therapeutic response to ATRA in APL patients. E5(−)E6(−) isoform of L-type fusion transcript with exons 5 and 6 deleted is associated with the ATRA-resistant phenotype

APL is also characterized by a specific chromosomal translocation (Fig. 2a) between the retinoic acid receptor alpha (*RARA*) and a number of fusion partners (*X-RARA*). This chromosomal rearrangement plays a critical role in the disease phenotype, particularly regarding ATRA sensitivity. Although a high proportion of APL patients achieve complete remission after treatment with ATRA, most patients who receive continuous ATRA treatment later relapse and develop the ATRA-resistant phenotype of this disease [30]. At least 98% of APL patients carry the t(15;17) translocation, resulting in *RARA* fusion with the promyelocytic leukemia (*PML*) gene (*PML-RARA)* [31]. The fusion of *PML* sequences to *RARA* regions increases fusion receptor affinity for co-repressors [32]. Therefore, the increased levels of ATRA are required to induce dissociation of co-repressors and to promote a therapeutic response to the treatment. In addition to *PML*, a limited number of patients exhibit a variety of other *X-RARA* fusions [33–39]. The fusion partner also plays a key role in the response to the retinoid treatment: APL patients carrying *NPM1* and *NuMA* fusion partners respond clinically to ATRA treatment [40, 41], whereas APL cases involving *PLZF* (promyelocytic leukemia zinc finger), *IRF2BP2* (interferon regulatory protein 2 binding protein 2) and *STAT5b* presented with ATRA resistance and a poor prognosis [42–45]. One of the most important tools in APL treatment is minimal residual disease monitoring with a special focus on the molecular detection of the *PML-RARA* transcript. Although the possibility of this monitoring was also reported in patients with *PLZF-RARA*- and *STAT5b-RARA*-positive diseases, no data regarding the clinical value of this tool are available [46, 47].

Molecular analysis of the possible mechanisms of retinoid resistance suggested that the reciprocal *RARA-PLZF* fusion product from the derivative chromosome 17 [der(17)] functions as a transcriptional activator targeting PLZF-binding sites, leading to cellular retinoic acid-binding protein 1 (CRABP1) upregulation. The CRABP1 protein is structurally similar to the cellular retinol-binding proteins, sequesters retinoic acid to limit its access to the nucleus [48], and is a well-established mediator of retinoid resistance in various biological models [49–51]. Similarly, APL patients expressing both fusion gene products exhibited primary resistance to ATRA [42, 52, 53]. In contrast, blast cells from a patient with the *PLZF-RARA* fusion transcript only were sensitive to ATRA treatment under in vitro conditions, and these results correlated with clinical remission after ATRA administration in this patient [54]. Moreover, two fusion proteins, PLZF-RARA and RARA-PLZF, negatively impacted the activity of CCAAT/enhancer binding protein α (C/EBPα), a master regulator of granulocytic differentiation [55]. Further research in a murine APL model demonstrated that the co-administration of 8-CPT-cAMP (8-chlorophenylthio-adenosine-3′, 5′-cyclic monophosphate) improves the therapeutic effect of ATRA by enhancing cellular differentiation and increasing PLZF-RARA degradation [56]. Nevertheless, the ability of this type of combined differentiation therapy to overcome retinoid resistance has never been proven in humans.

Published results on APL cell lines also suggest a possible association between the splicing variants of the *PML-RARA* fusion gene and the therapeutic response to ATRA [57]. These variants resulted from the alternative splicing of the *PML* sequence, which contains heterogeneous breakpoint cluster regions (bcrs) at three different sites (Fig. 2b) [58–60].

Sequencing analysis of the *PML-RARA* gene in a cohort of 79 APL patients showed that the L-type fusion transcript resulting from the alternative splicing was present in three isoforms. One of these isoforms, the E5(−)E6(−) isoform with exons 5 and 6 deleted, is associated with the ATRA-resistant phenotype [57]. A subsequent localization study reported that the E5(−)E6(−) protein was detected in the cytoplasm only, whereas the other two isoforms were distributed throughout the nucleus and cytoplasm. The exclusive cytoplasmic localization of the E5(−)E6(−) isoform is apparently responsible for inhibiting ATRA-dependent transcription and for subsequently blocking cell differentiation. Thus, monitoring E5(−)E6(−) isoform expression in APL patients with the L-type *PML-RARA* fusion gene might be helpful for predicting a patient’s response to ATRA treatment.

Similarly, APL cells with the V-type splicing isoform, characterized by exon 6 truncation, were also reported to be less sensitive to ATRA treatment. In this group of APL patients, a subset with lower ATRA sensitivity presented with a relatively long “spacer” with a cryptic coding sequence inserted into the joining sites between the truncated *PML* and *RARA* mRNA fusion partners. Subsequent in vitro studies confirmed these results, revealing that spacer deletion restored ATRA sensitivity [61].

### Predictive biomarkers in pancreatic ductal adenocarcinoma

The vitamin A metabolism disturbances that result in a decreased intracellular ATRA concentration were originally described in pancreatic ductal adenocarcinoma (PDAC) [62] and later, in other human malignancies, also [63]. Previous studies in PDAC cell lines have indicated the ability of ATRA to induce cell cycle arrest and differentiation, although these data revealed highly variable retinoid sensitivity among the PDAC cell lines [64, 65]. Based on the receptor-dependent retinoid mechanism, the potential patient benefit from this treatment is highly dependent on the retinoid receptor expression level in tumor tissue. Among others, *RARβ* expression is downregulated in PDAC [66–68], which may explain the negative outcomes of clinical trials focused on retinoid treatments.

ATRA typically induces cell differentiation and growth arrest in most epithelial cell types. However, experiments in Capan-1 cell line have shown that in addition to an antiproliferative effect, retinoids increase cell migration, resulting in an invasive phenotype [69]. This effect is probably caused by the presence of the nuclear receptors PPARβ/δ, which are also activated by exogenous retinoids and form heterodimers with RXR. While canonical RAR-dependent gene expression leads to growth arrest, PPARβ/δ activation initiates proliferation, cell survival and tumor growth in mouse model [70]. The distribution of available ATRA between PPARβ/δ and RAR receptors is regulated by the levels of two key intracellular ligand-binding proteins: fatty acid-binding protein 5 (FABP5) and cellular retinoic acid-binding protein 2 (CRABP2). Depending on their relative abundance within the cell, FABP5 and CRABP2 transport exogenous retinoids from the cell cytoplasm into the nucleus, to either PPARβ/δ or RARs [17]. A recent study on 14 PDAC cell lines demonstrated that it might be possible to predict PDAC cell sensitivity to ATRA on the basis of the relative expression levels of these two retinoid-binding proteins. According to this study, 10 of 14 cell lines expressed the one or the other binding protein confirming the pattern of reciprocal differential expression of both transcripts in PDAC cells. FABP5^high^CRABP2^null^ PDAC lines were resistant to ATRA-mediated growth inhibition and apoptosis and also exhibited an increased migration and invasion phenotype. In contrast, FABP5^null^CRABP2^high^ cell lines retained ATRA sensitivity. These results were also confirmed in vivo using xenograft models [71]. Immunohistochemical detection of FABP5 in PDAC samples revealed that about 20% of them were completely negative for FABP5 indicating these patients as suitable candidates for retinoid therapy [71]. Since the retinoid binding affinity of the CRABP2-RAR pathway is higher than that of the FABP5-PPARβ/δ pathway, at least a partial ATRA-mediated tumor-suppressive effect is expected in tumors with comparable FABP5 and CRABP2 expression.

### Predictive biomarkers in breast carcinoma

Breast carcinoma is a heterogenous disease classified into subtypes according to the expression of biological markers, such as estrogen receptor (ER), progesterone receptor (PR) and epidermal growth factor receptor 2 (HER2) [72–74]. According to recent clinical trials aimed at investigating the efficacy of retinoids as adjuvant treatment in breast carcinoma, some patients benefited from the retinoid treatment. Moreover, the breast carcinoma cell response to retinoids can be predicted by evaluating the expression of several marker proteins.

Indeed, several studies have demonstrated that the average RARα receptor level is significantly higher in ATRA-sensitive than ATRA-resistant breast carcinoma cell lines [75–77]. Furthermore, a truncated RARβ’ isoform has also been identified in some of these cell lines and it has been associated with increased cell proliferation and ATRA resistance [78].

Another potential marker of ATRA resistance was suggested by a study describing Her2/neu-induced ATRA resistance in breast cancer cell lines [79]. *ERBB2* transfection in ATRA-sensitive breast carcinoma cells induced ATRA resistance. When Her2/neu was blocked by trastuzumab, the cells exhibiting induced ATRA resistance became ATRA sensitive again. This study also hypothesized that Her2/neu may induce ATRA resistance in breast carcinoma cells by suppressing RARA expression and/or by deregulating the G1 checkpoint of the cell cycle.

As described in the PDAC section in this review, the abundance of the intracellular retinoic acid transporters CRABP2 and FABP5 within the cell can indicate breast carcinoma cell response to ATRA, since these molecules have been shown to play opposing roles in mediating the cellular response to retinoids [17]. According to the microarray analysis of gene expression in 176 primary breast carcinoma samples, *FABP5* is preferentially upregulated in estrogen receptor-negative (ER-) and triple-negative breast carcinoma cells (TNBC), and an increased *FABP5* mRNA level is associated with poor patient prognosis and high tumor grade [80]. In this study, *FABP5* normalized signal intensity scores were categorized into high versus low using cut-off point of 0.768. In this cohort, 61% of patients showed high *FABP5* expression and these patients had a significantly decreased survival rate if compared with those with low *FABP5* expression. Moreover, *FABP5* silencing in Hs578T breast carcinoma cell line resulted in approximately 40% reduction in proliferation activity. However, although breast cancer cells with an increased FABP5/CRABP2 ratio present with increased ATRA resistance, this ratio does not always accurately predict the breast cancer cell response to ATRA, indicating that other factors are also involved in the mechanism of retinoid resistance development. Another recent study identified CRABP1 as the third key player that potentially influences the breast cancer cell response to ATRA. This protein has been identified as a retinoid inhibitor and probably sequesters retinoic acid in the cytoplasm, thereby preventing RAR activation in the nucleus. Similarly to FABP5, CRABP1 is also preferentially expressed in ER- and TNBC tumor tissues that are prone to ATRA resistance [81]. According to this study, CRABP1 synergizes with FABP5 to compete with CRABP2 for retinoic acid molecules, thereby reducing retinoic acid access to RARs within the nucleus.

These findings provide molecular tools to predict and eventually overcome ATRA resistance in breast carcinoma therapy. CRABP1 and FABP5 co-expression may serve as a predictive biomarker of ATRA resistance in this tumor type, and the downregulation may be a key step in (re)sensitizing breast carcinoma cells to retinoid therapy. A novel mechanism for resensitizing ATRA-resistant cells to ATRA-mediated apoptosis was recently introduced: the phytochemical curcumin is able to upregulate CRABPII, RARβ and RARγ expression in TNBC cell lines and thereby sensitizes cells to ATRA-induced apoptosis. This reversed resistance to ATRA-induced apoptosis in TNBC cells was dependent on the curcumin dose and treatment length [82]. Overall, this study highlights the potential of curcumin as a possible therapeutic adjuvant in ATRA-resistant breast carcinomas.

Another recent study compared the phosphoproteome and transcriptome of established ATRA-sensitive and ATRA-resistant cell lines derived from breast carcinoma (MCF7, BT474). One of the most interesting results was that ATRA did not regulate the phosphorylation of the same proteins in both cell lines, i.e., the ATRA-resistant cell line exhibited a deregulated kinome. High-throughput sequencing experiments revealed that 80% of the genes regulated by ATRA in MCF7 cells were not regulated in BT474 cells and vice versa. Additionally, 40% more genes were regulated by ATRA in the MCF7 cells than in the BT474 cells. Moreover, this study indicates that ATRA induced RARα phosphorylation in resistant cell lines only, which may cause kinome deregulation and consequences in other intracellular metabolic pathways [83].

### Predictive biomarkers in neuroblastoma

Neuroblastoma (NBL) is a neuroectodermal tumor arising from elements of the neural crest and represents the most common extracranial solid tumor in children. In a subset of high-risk NBL patients with minimal residual disease, retinoid administration was proven effective as a part of postconsolidation therapy after intensive multimodal treatment. Unfortunately, approximately 50% of this patient population is resistant to this treatment or develops resistance during therapy [84]. Moreover, a recent study evaluated the efficacy and safety of additional retinoid therapy in NBL patients and presented a more critical view, concluding that no clear evidence exists for a difference in overall survival and event-free survival in patients with high-risk NBL treated with or without retinoids [85]. However, the usefulness of differentiation therapy with retinoids largely depends on the ability to identify a subset of NBL patients who benefit from this treatment, according to analyses of retinoid resistance/sensitivity markers. Recent investigations on the mechanisms of retinoid resistance identified several downstream retinoid-regulated proteins and discussed these proteins as possible predictive biomarkers for the clinical response to retinoid treatment.

PBX1 belongs to the three-amino-acid loop extension (TALE) family of atypical homeodomain proteins and interacts with other homeodomain-containing nuclear proteins, such as HOX and MEIS, to form heterodimeric transcription complexes. PBX1 is involved in a variety of biological processes including cell differentiation and tumorigenesis [86, 87]. Recent study revealed that in NBL cell lines treated with 13-*cis*-RA, PBX1 mRNA and protein expression levels are both induced in 13-*cis*-RA-sensitive cell lines only. After treatment with 13-*cis* RA, all 6 RA-sensitive cell lines showed a significant increase in *PBX1* expression, whereas RA-resistant cell lines exhibited no such effect. These studies also revealed that reduced PBX1 protein levels result in an aggressive growth phenotype and 13-*cis*-RA resistance. Finally, the authors demonstrated that in primary NBL tumor tissue, PBX1 expression correlated with the histological NBL subtype, with the highest PBX1 expression in benign ganglioneuromas and the lowest expression in high-risk NBL [88].

Homeobox (HOX) proteins function as regulators of morphogenesis and cell fate specification and are key mediators of retinoid action in nervous system development. Among members of the HOX family of transcription factors, HOXC9 seems to play an important role in neuronal differentiation. A recent study revealed that the *HOXC9* promoter is epigenetically primed in an active state in ATRA-sensitive NBL cell lines and in a silenced state in ATRA-resistant NBL cell lines. Moreover, HOXC9 protein levels were significantly higher in differentiated NBL cells than in NBL cells undergoing ATRA-induced differentiation [89].

The protein neurofibromin 1 (NF1) is known to antagonize the activation of RAS proteins but is also involved in other signaling pathways, such as the cAMP/PKA pathway [90]. NF1 controls the retinoid treatment response in NBL cells through the RAS-MEK signaling cascade and has been identified as the lead candidate gene for influencing retinoic acid-induced differentiation in NBL cell models [91]. According to this study, SH-SY5Y cells with *NF1* knockdown continued to proliferate when exposed to RA in contrast to the control cells. Subsequent experiments showed downregulation of RA target genes in *NF1* knockdown cells. These results may indicate the role of NF1 in maintaining RA resistant phenotype.

In further research, genomic aberrations of the *NF1* gene were found in 6% of primary NBL representing a subset of cases where the loss of *NF1* gene could be caused by gene mutation.

A connection between NF1-RAS-MEK signaling and retinoic acid action was demonstrated by the finding that the NF1-RAS-MEK cascade suppresses ZNF423 protein expression, which functions as a RAR/RXR coactivator. Additionally, tumors with activated RAS signaling and low ZNF423 expression present with a poor response to 13-*cis*-RA (isotretinoin) treatment. Moreover, decreased *NF1* and *ZNF423* gene expression, reflecting hyperactivated RAS/MAPK signaling, is correlated with a very poor clinical outcome in NBL patients and was detected in 78% of patients with relapsed NBL [92], whereas high expression levels both of these proteins are associated with the best prognosis in NBL patients. As a result, Holzel and colleagues suggest that pharmacological MEK inhibition can sensitize NBL cells that are resistant to retinoid-induced terminal differentiation. Although these data seem to be readily translatable, several important questions will need to be addressed before incorporating this therapy into clinical practice. It will be critically important to determine how MEK inhibition combined with isotretinoin will fit into the overall NBL treatment strategy and whether MAPK pathway activation is a mechanism of acquired resistance to isotretinoin therapy or a collateral event of oncogenic driver mutations only [93]. Another recent study also indicated a potential role of MEK cascade inhibition in overcoming ATRA resistance in malignant peripheral nerve sheath tumors (MPNST) in vitro, but no correlation was found between *ZNF423* mRNA levels and the sensitivity of MPNST cells to ATRA [94]. These results demonstrate that some other mechanisms are involved in maintaining ATRA resistance of MPNSTS cells.

High-mobility group A (HMGA) proteins function as ancillary transcription factors and regulate gene expression through direct DNA binding or protein-protein interactions and play important functions in controlling cell growth and differentiation. *HMGA2* is completely absent in adult organisms; its expression is restricted to rapidly dividing embryonic cells and tumors with epithelial and mesenchymal origins [95]. *HMGA2* was also detected in some retinoid-resistant NBL cell lines. In NBL cell lines, a causal link between *HMGA2* expression and retinoid-induced growth arrest inhibition was proven using exogenous *HMGA2* expression, which was sufficient to convert *HMGA2*-negative, retinoid-sensitive cells into retinoid-resistant cells [96]. In contrast, *HMGA1* was found to be expressed at different levels in all NBL cell lines [97], indicating that its action is necessary for functions conserved throughout the developmental differentiation of the sympathetic system.

UNC45A, another potential marker of retinoid resistance, is a protein encoded by the *UNC45A* gene, a member of *UNC45*-like genes, which are evolutionarily highly conserved, and the resulting protein products are involved in muscle development and myosin assembly [98]. The UNC45A protein has been shown to modulate the HSP90-mediated molecular chaperoning of the progesterone receptor, since the UNC45A blocks the chaperoning of this receptor to the hormone-binding state [99]. In NBL cell lines, the role of UNC45A in causing ATRA resistance was suggested by Epping and co-workers [100]. When UNC45A was ectopically expressed in their experiments, ATRA-sensitive human NBL cell lines failed to undergo growth arrest after ATRA treatment. The UNC45A protein levels required for ATRA resistance were similar to the levels in several cancer cell lines. Neither the endogenous nor the ectopically expressed UNC45A protein levels were affected by ATRA treatment. Moreover, UNC45A expression also inhibited the differentiation of NBL cells cultured in the presence of ATRA, indicating the resistant phenotype.

## Conclusion

This review was aimed to summarize the current knowledge, both clinical and experimental, on predictive markers in human cancers that are treated with retinoids as a part of the therapeutic regimen. This review demonstrated that each described cancer type seems to have a unique pattern of altered signaling pathways, resulting in a set of predictive biomarkers that indicate retinoid resistance or sensitivity, which is typical for this malignancy. Many of the research studies mentioned in this review are only initial, and the acquired results require further detailed investigation and clinical validation of the proposed predictive biomarkers. However, these studies demonstrate the promising future for differentiation therapies that use retinoids, especially in identifying reliable markers that predict the response of each individual patient to this type of treatment. Hopefully, the personalized approach will be a new milestone in anticancer differentiation therapy.

## Abbreviations

13-*cis*-RA: 13-*cis* retinoic acid
4-HPR: Fenretinide (N-(4-hydroxyphenyl) retinamide)
8-CPT-cAMP: 8-chlorophenylthio-adenosine-3′, 5′-cyclic monophosphate
9-*cis-*RA: 9-*cis* retinoic acid
AML: Acute myeloid leukemia
APL: Acute promyelocytic leukemia
ATO: Arsenic trioxide
ATRA: All-*trans* retinoic acid
BC: Breast carcinoma
Bcr: Breakpoint cluster region
C/EBPα: CCAAT/enhancer binding protein α
CRABP: Cellular retinoic acid-binding protein
ER: Estrogen receptor
FABP5: Fatty acid-binding protein 5
FDA: Food and Drug Administration
GCSF: Granulocyte-colony stimulating factor
HER2: Epidermal growth factor receptor 2
HMGA: High-mobility group A
HOX: Homeobox
IRF2BP2: Interferon regulatory protein 2 binding protein 2
MN1: Meningioma 1
MPNST: Malignant peripheral nerve sheath tumor
NBL: Neuroblastoma
PDAC: Pancreatic ductal adenocarcinoma
PLZF: Promyelocytic leukemia zinc finger
PML: Promyelocytic leukemia
PPAR: Peroxisome proliferator-activated receptor
PR: Progesterone receptor
RAR: Retinoic acid receptor
RARE: Retinoic acid response elements
RXR: Retinoid X receptor
TALE: Three-amino-acid loop extension
TNBC: Triple-negative breast cancer
